# Characterization and Functional Analysis of Polyadenylation Sites in Fast and Slow Muscles

**DOI:** 10.1155/2020/2626584

**Published:** 2020-03-16

**Authors:** Lulu Deng, Long Li, Cheng Zou, Chengchi Fang, Changchun Li

**Affiliations:** Key Lab of Agriculture Animal Genetics, Breeding, and Reproduction of Ministry of Education, College of Animal Science and Technology, Huazhong Agricultural University, Wuhan 430070, China

## Abstract

Many increasing documents have proved that alternative polyadenylation (APA) events with different polyadenylation sites (PAS) contribute to posttranscriptional regulation. However, little is known about the detailed molecular features of PASs and its role in porcine fast and slow skeletal muscles through microRNAs (miRNAs) and RNA binding proteins (RBPs). In this study, we combined single-molecule real-time sequencing and Illumina RNA-seq datasets to comprehensively analyze polyadenylation in pigs. We identified a total of 10,334 PASs, of which 8734 were characterized by reference genome annotation. 32.86% of PAS-associated genes were determined to have more than one PAS. Further analysis demonstrated that tissue-specific PASs between fast and slow muscles were enriched in skeletal muscle development pathways. In addition, we obtained 1407 target genes regulated by APA events through potential binding 69 miRNAs and 28 RBPs in variable 3′ UTR regions and some are involved in myofiber transformation. Furthermore, the de novo motif search confirmed that the most common usage of canonical motif AAUAAA and three types of PASs may be related to the strength of motifs. In summary, our results provide a useful annotation of PASs for pig transcriptome and suggest that APA may serve as a role in fast and slow muscle development under the regulation of miRNAs and RBPs.

## 1. Introduction

Polyadenylation involving in the cleavage of 3′ signaling region of precursor mRNA (pre-mRNA) and the addition of a poly(A) tail is the final core step of mRNA maturation [1, 2]. The upstream and downstream cis elements constantly regulate the cleavage of polyadenylation sites (PASs). These elements mainly consist of an upstream canonical poly(A) signal AAUAAA and a less defined downstream U/GU-rich region (typically GUGU) [3, 4]. Alternative polyadenylation (APA), a phenomenon that the same RNA molecule with distinct 3′ terminal, is widespread in eukaryotes as an important posttranscriptional regulatory mechanism. More than 70% of human genes produced mRNA isoforms by APA and about half in Arabidopsis, *Caenorhabditis elegans*, and zebrafish [5–8]. APA events can be classified roughly into two categories. In one case, PASs located in internal exons or introns named coding region-APA (CR-APA) affect the length of coding regions. In another case, PASs located in the 3′ untranslated region (3′ UTR) (UTR-APA) result in the production of transcripts with diverse 3′ UTRs [9].

The development of skeletal muscles after being born is characterized by hypertrophy and type transformation of muscle fibers. Factors such as exercise, nutrition, and aging can lead to the transformation of four muscle fibers (I, IIA, IIX, and IIB) [10–13]. The fast and slow muscles contain four different muscle fibers in different proportions, resulting in differences in morphological, physiological, and biochemical characteristics [14, 15]. APA events can regulate muscle fiber transformation of fast and slow muscles and have gained more attention in recent years. Ocular pharyngeal muscular dystrophy (OPMD) is mainly expressed in myasthenia and amyotrophy of the eye and pharyngeal muscles [16]. de Klerk et al. found that an effect of poly(A) binding protein nuclear 1 (PABPN1) on APA may partly explain muscle weakness in OPMD [17]. Subsequently, a further study observed that the distal PAS utilization rates of six genes (Atrogin-1, Arih2, Psme3, Psmd14, Rad23a, and Atg12) were significantly downregulated in PABPN1-DR mouse model. Reduced PABPN1 levels may cause Atrogin-1 dysregulation and also gave rise to myofiber atrophy, ECM thickening, and myofiber transitions [18]. In addition, the loss or gain of PASs in AMPK*α*1KO cells and AMPK*α*1KO mice resulted in changes in muscle area and enrichment of five important differentially expressed genes (Car3, Mylk4, Neb, Obscn, and Pfkm) and further regulated muscle development [19]. For example, Nebulin is a large actin-binding protein and the main cause of nemaline myopathy (NEM) [20].

Next-generation sequencing (NGS) technology has been widely used for transcriptome research on regulatory mechanisms and differential gene expression [21]. However, the length of sequencing reads limits the reconstruction of full-length splice variants [22]. And it is difficult to separate genuine PASs from internal priming events [23–25]. Based on single-molecule real-time (SMRT) sequencing, transcripts derived from PacBio platform without assembly are longer and even full length, which provide direct evidence for the annotation of isoforms of each gene. Especially, the Iso-seq library built with the oligo(dT) primer ensures the integrity of 3′ terminal. So far, the PacBio protocol Iso-Seq™ has been successfully applied to characterize the posttranscriptional APA events in various eukaryotes [26–30]. Our study used the PacBio dataset to annotate PASs of pigs and analyzed differential usages and functions of APA genes under the regulation of miRNAs and RBPs between fast and slow muscles. We also discussed the distribution of cis elements surrounding all identified PASs. Thus, this study updated pig genome annotation about PASs and supplied valuable resources for further transcriptome research.

## 2. Materials and Methods

### 2.1. Ethics Approval and Consent to Participate

The experimental white pigs were provided by the National Livestock Engineering Research Center of Huazhong Agricultural University. Animal breeding and slaughtering were carried out in accordance with the preapproval guidelines of the Standing Committee of the Hubei Provincial People's Congress No. 5. And all the experimental schemes were approved by the Scientific Ethics Committee of Huazhong Agricultural University.

### 2.2. Data Generation

The PacBio datasets in this paper were from our group to collect 38 tissues of Large White, including one organ (a 26-day-old whole embryo), 20 tissues (heart, kidney, tongue, stomach, liver, spleen, lung, longissimus muscle, duodenum, cecum, inguinal lymph nodes, precaval vein blood, inner ear, back subcutaneous fat (BSF), ovary, psoas muscle, endometrium (EN), corpus luteum, soleus muscle (SM), and extensor digitorum longus (EDL)) from an adult sow (210 days of age), and 17 tissues (heart, tongue, stomach, uterus, liver, spleen, lung, longissimus muscle, duodenum, inguinal lymph nodes, precaval vein blood, BSF, psoas muscle, EN, SM, thymus, and EDL) from a one-day-old sow [31]. The total RNA of each tissue was extracted and pooled together.

Besides, RNA-seq datasets were also derived from data generated by paired-end sequencing of 8 tissues (BSF, SM, EDL, and EN from adult and one-day-old sows) on the illumine HiSeq2500 platform among 38 tissues described above [31].

### 2.3. Identification of PASs

By annotating loci and isoform, and removing redundant and false-positive gene structures, we constructed a gene structure annotation for FLNC reads. The specific method was performed as previously described [31]. Our Iso-Seq libraries were constructed using an oligo dT primer, and 3′ end of each isoform was retained. We used Transcriptome Analysis Pipeline for Isoform Sequencing (TAPIS) pipeline according to the default options to identify PASs. The site with maximum read depth was considered as a genuine PAS if the distance between multiple candidate sites was within 15 nt.

### 2.4. Motif Analysis

To assess the sequence composition surrounding cleavage sites, we calculated the proportion of each nucleotide at each position within the ±100 nt window. The upstream and downstream of PASs usually contain canonical recognition signals and other hexamer variants. We performed de novo motif searches using Signal Sleuth (v2) program with Motif Overlapping mode for hexamers that appeared in a 100 nt window upstream and downstream and a 50-nucleotide window upstream of identified PASs [32]. In addition, based on the single or multiple ends of PASs in the 3′ UTR region, these PASs were classified into three types: single end, single-end PASs that found in genes with single terminal 3′ UTR isoform; distal, distal PASs regarded as the last PASs of terminal 3′ UTR that undergo APA events; and proximal, proximal PASs defined as all PASs of terminal 3′ UTR that undergo APA events except the distal one. Nucleotide distributions of these three types were calculated and shown by Weblogo3 (v3.6) with the default color scheme [33]. Then, the Find Individual Motif Occurrences (FIMO, v5.0.2) method with *p* value <0.001 of MEME suite was utilized to calculate the frequency of the canonical polyadenylation signal AAUAAA and 12 most common variants (AUUAAA, AAUAUA, UAUAAA, AGUAAA, AAUACA, UUUAAA, AAAACA, CAUAAA, AAGAAA, GAUAAA, AAAAG, and AAUAGA) reported in the upstream 50-nucleotide window of three types of PASs [34, 35].

### 2.5. Tissue-Specific Analysis

After filtering out of adaptor sequences and discarding low-quality reads in RNA-seq raw data using Trimmomatic (v0.36), clean reads were mapped to the reference genome (ftp://ftp.ensembl.org/pub/release-94/fasta/sus_scrofa/dna/) of Sus scrofa with Hisat2 (v2.0.3) [36, 37]. Then, we used StringTie (v1.3.4) to reconstruct the transcriptome for each sample on the basis of the structure annotation file above, and then all transcriptomes were merged into one nonredundant transcriptome using StringTie merge function [38]. We finally obtained PASs located in each sample by comparing positions of assembled transcripts with all identified PASs. The average length of 3′ terminal in each tissue was replaced by the average of 3′ UTR lengths of all genes. Moreover, the number of reads mapping to PAS-associated genes was counted using the htseq-count program (v0.6.1) and normalized as the gene expression [39]. Default parameters were used in all abovementioned programs. Because the lack of expression in PacBio transcriptome sequencing limits our quantitative and differential analysis of PASs coexpressed in each group, we define differential PASs as PASs expressed only in one group and not in other groups.

### 2.6. Prediction of Binding Sites about miRNAs and RBPs

PASs located in genes with annotated START and STOP codons were selected from all 3′ UTR PASs. In case where multiple STOP codons appeared, we determined coordinates of the codons that minimized the 3′ UTR length as the only STOP codons. Extended 3′ terminals of isoforms of PAS-associated genes were globally searched for the targeted binding sites of reported 457 miRNA mature seed sequences and 64 RBPs motif using Analysis of Motif Enrichment (AME, v5.0.3) method of the MEME suite [40]. Then, we selected differential miRNA and RBP binding sites based on APA genes. The motifs of these RBPs were obtained from the CisBP-RNA database (http://cisbp-rna.ccbr.utoronto.ca/bulk.php), and the seed sequences of the miRNAs were downloaded from the miRBase database (http://www.mirbase.org/cgi-bin/browse.pl).

### 2.7. Gene Ontology and Pathway Analysis

We performed Database for Annotation, Visualization, and Integrated Discovery (DAVID) analysis to categorize the considerably enriched (*p* < 0.05) biological processes or pathways to assess the properties of genes with differential expression of PASs or variable presence of miRNAs and RBPs [41]. Because of the limitation of the Sus scrofa genome annotation in the DAVID database, we converted all genes into human homologous genes by BIOMART from Ensembl.

### 2.8. Validation of Variable 3′ End by 3′ RACE

We randomly selected 6 APA genes in porcine skeletal muscle for experimental validation as described in other articles [28, 30]. The cDNA was prepared by reverse transcription of SMART Scribe TM Reverse Transcriptase (100 U). Next, 2.5 *μ*L of cDNA was amplified using the cDNA ends with gene-specific primers (GSPs) and Seq Amp DNA Polymerase. All GSPs for validation are listed in [Supplementary-material supplementary-material-1].

## 3. Results

### 3.1. Identification and Annotation of PASs

**Figure 1:**
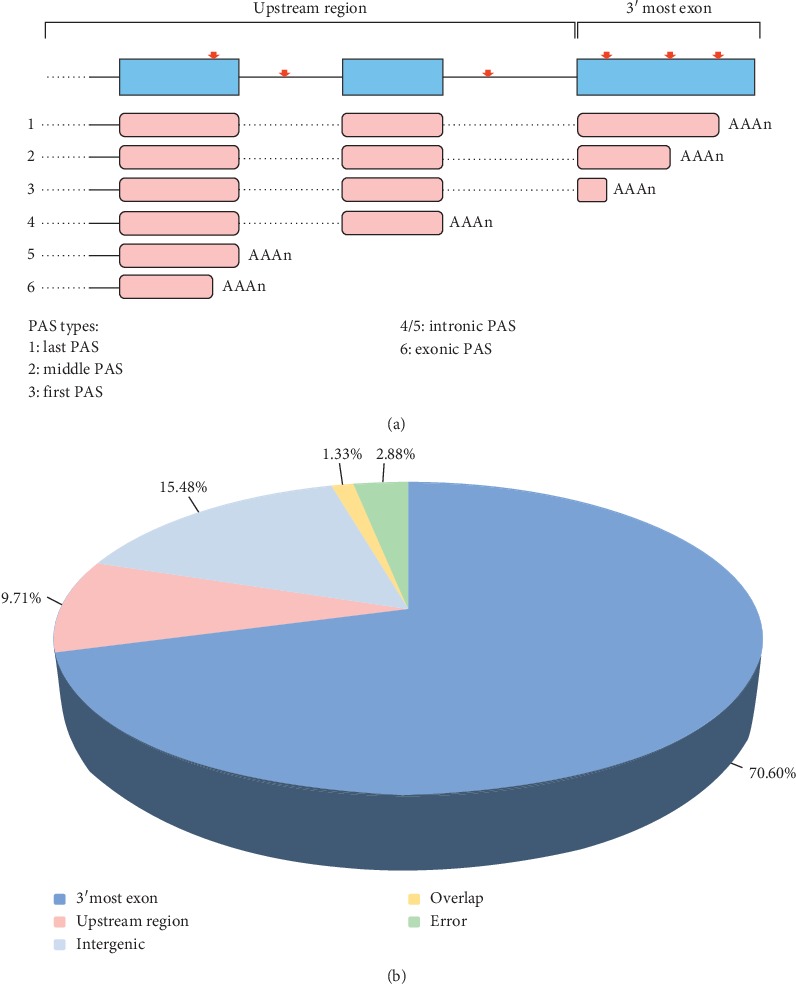
Schematic about PAS types. (a) Two PAS types across genomic regions including PASs in upstream region and 3′ most exon. The type number corresponded to isoform numbers shown in the figure. Blue and pink boxes, exons. Solid lines, introns. Red arrows, cleavage. Dashed lines, splicing. (b) Annotation and distribution of PASs from Ensembl. Intergenic, PASs in intergenic. Overlap, PASs in overlap genes. Error, PASs located in misannotated gene.

Our research group has established an Iso-Seq database upon 38 Large White pig tissues [31]. Based on that, we used the TAPIS pipeline to identify PASs [30]. A total of 10,334 PASs were obtained, of which 5 identified on chromosome Y were removed. And other PASs were located in 6668 genes. Each gene was supported by an average of 37 poly(A) reads. Comparing with the Ensembl reference annotation, 84.52% of PASs were aligned to the annotated genes, and the remaining 1600 PASs were mapped to the intergenic regions (Figure 1(b) and [Supplementary-material supplementary-material-1]), which might represent unknown genes or novel distal sites in the downstream 3′ UTR regions of the known genes. These PASs which were located in annotated genes mainly corresponded to two modes based on the annotated genomic features: 3′ most exon and upstream region (Figure 1(a)). As expected, major (83.52%, 7295) PASs were in the type of 3′ UTR regions, and a small part of PASs was in introns (9.61%, 839), CDS (1.59%, 139), and 5′ UTR regions (0.29%, 25). Considering that PASs in genomic regions only belonged to a small proportion of the overall polyadenylation events, we mainly concentrated on PASs that were located in 3′ most exon. However, we found that a portion of genes might be misannotated for no match with our PacBio isoforms. In the first scenario, some adjacent annotated genes in reference annotation overlapped only a single gene in our structural annotation ([Supplementary-material supplementary-material-1]), and another scenario was contrary to the first ([Supplementary-material supplementary-material-1]). Thus, we removed PASs in those misannotated genes and some other PASs (138) located in overlapping genes in the following analysis.

### 3.2. Distribution of PASs in the Pig Genome

**Figure 2:**
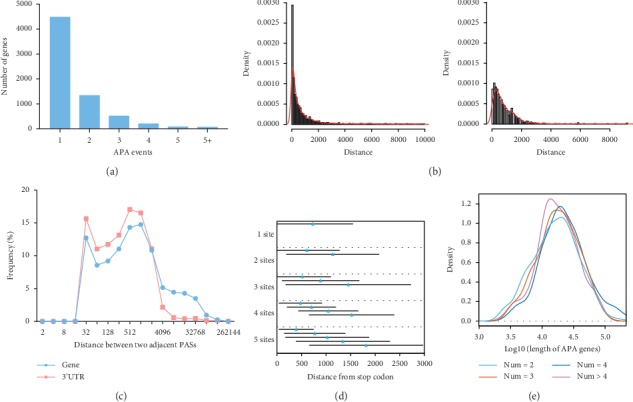
Distribution of PASs in APA genes and 3′ UTR regions. (a) Number of genes containing one or more PASs. (b) Histogram of the genomic distances between two adjacent PASs in APA genes and 3′ UTR regions. (c) Frequency of the genomic distances between two adjacent PASs in APA genes (pink) and 3′ UTR regions (powder blue). (d) Blue triangle, the average distance between the PAS and corresponding stop exon in 3′ UTR regions. Black horizontal solid line, standard deviation of the average distance. (e) Density of length for APA genes with different number of poly(A) sites.

All identified PASs were used to analyze the distribution of PASs in genes. Among 6668 PAS-associated genes, 2191 (32.86%) contained two or more polyadenylation events (APA) which resulted in variable 3′ terminal (Figure 2(a)), and with an average of 1.55 PASs per gene. The distances between adjacent sites in each APA gene and their 3′ UTR regions were calculated. We observed that most of them were relatively near in both cases, but presented two different modes obviously (Figure 2(b)). More than 75% and 69% of APA were shorter than 1 kb (Figure 2(b)). Then, our further analysis showed that the first two peaks of gene regions were similar to those of 3′ UTR with a higher ratio, but the last peak that was mainly caused by the location of more PASs in the intron was only found in the gene regions (∼15 kb) (Figure 2(c)). Then, we plotted the average position of PASs in 3′ UTR regions according to the stop codon for numbers of PASs. Although the position distribution of PASs showed great changes, the distance at the same position indicated a slightly shortened trend as numbers of PASs in 3′ UTR genes are increasing (Figure 2(d)). Finally, the length of APA genes was discovered to be related to the number of PASs as shown in Figure 2(e), which was consistent with previous studies in moso bamboo [28].

### 3.3. The Usage of Upstream and Downstream Elements

**Figure 3:**
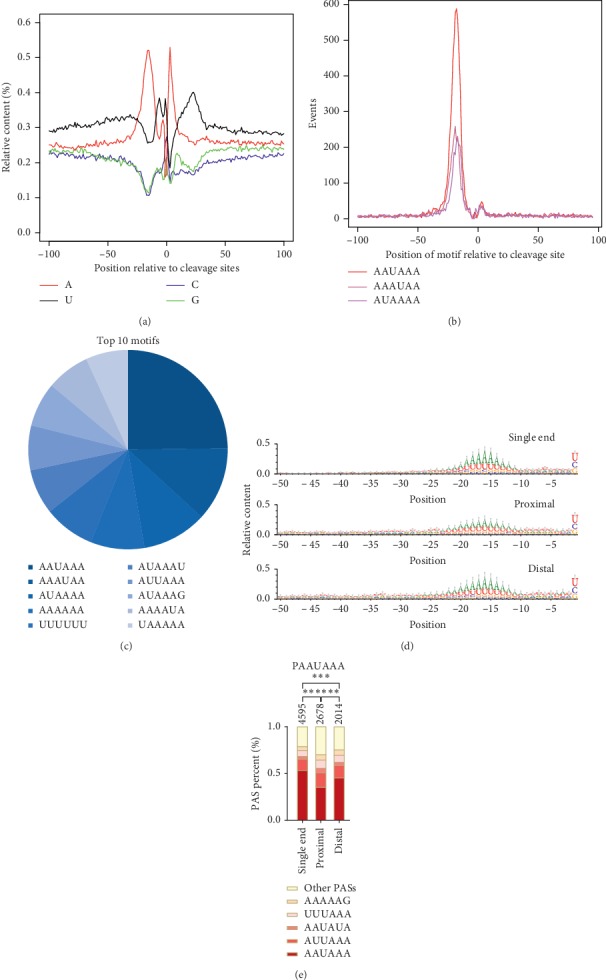
Characteristics of elements and properties of different types of PASs. (a) Plotted the nucleotide distribution surrounding all identified PASs in a ±100 nt window. (b) Enrichment of the top three hexamers surrounding all identified PASs in a ±100 nt window. (c) The top 10 hexamers surrounding all identified PASs in a 50 nt window upstream. (d) Plotted the nucleotide distribution surrounding three types (as described in the Method section) of PASs in a 50 nt window upstream. The vertical axis showed the ratio of nucleotides. (e) Percentages of the top 10 hexamers in the 50 nt window upstream of different PASs.

It has been well documented that PASs were involved in the core polyadenylation upstream signal and the U/GU-rich downstream sequence element [3, 7, 35]. We studied the sequence composition surrounding all PASs and observed the distribution of these two elements in our data (Figure 3(a)). Then, we performed de novo motif searches by using the ±100 nt sequences upstream and downstream of all PASs and detected a strong enrichment of canonical motif AAUAAA and other variant signals peaking at 20 nt position (Figure 3(b)). Furthermore, the statistical results of the top ten motifs in the 50 nt window upstream of these PASs are shown in Figure 3(c). The canonical motif and variants accounted for the majority as expected, while we observed unusual A and U enriched hexamer.

The usage of upstream elements usually affects the cleavage and polyadenylation. Although we found a similar nucleotide distribution among the three types of PASs, AU enrichment level upstream of the proximal sites was relatively lower compared to the other two (Figure 3(d)). We further calculated the canonical signal and other 12 most common variants [35] and observed that AAUAAA accounted for a large proportion within single-end PASs (Figure 3(e)). Amongst PASs that underwent APA events, we also found a similar but lower enrichment level of canonical signal in the distal PAS (Fisher's exact test, 45%, *p*=3.59*E* − 09). However, compared with distal PASs, proximal PASs obviously carried a significantly higher proportion of other weak variants with lower canonical signal (35%, *p*=1.92*E* − 12).

### 3.4. Tissue Specificity Analysis of PASs

**Figure 4:**
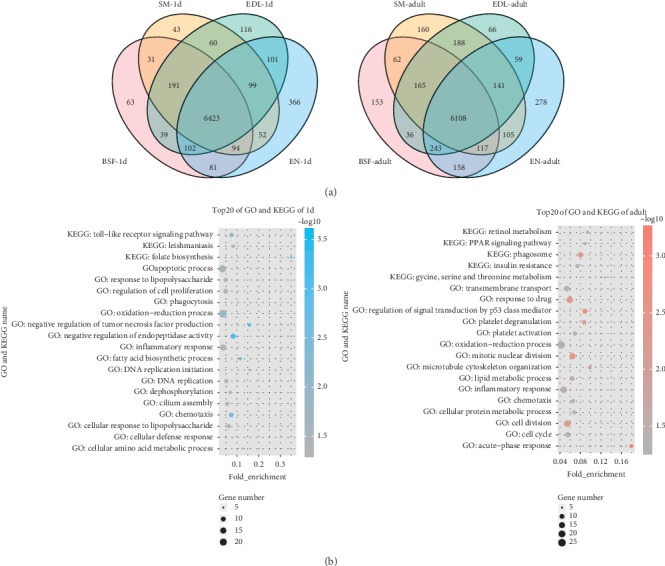
The usage of tissue-specific PASs in SM and EDL. (a) The number represented tissue-specific PASs identified in BSF, SM, EDL, and EN of 1 d and adult. (b) List of first 20 pathways and biological processes about the different usages of PAS-associated genes in two periods.

The tissue specificity of PASs has been confirmed in other organisms [8, 42, 43]. We analyzed PASs in four tissues at two ages by using RNA-seq datasets and the new modified structure annotation file. 6993, 7131, 7025, and 7319 PASs have be found in SM, EDL, SBF, and EN of 1 d and 7046, 7006, 7042, and 7210 PASs in adult tissues, respectively (Figure 4(a) and [Supplementary-material supplementary-material-1]). A total of 6423 and 6108 PASs had significant hits in both groups. And EN occupied the largest number of specific PASs that uniquely expressed. Moreover, we performed a further comparative analysis of tissue-specific PASs between SM and EDL in 1 d and adult periods (366 and 278), which were often used as representative muscles dominated by slow-twitch (type I) and fast-twitch (type II) fibers in animal experiments[44]. Gene ontology analysis revealed that these genes containing tissue-specific PASs (*p* < 0.05) were mainly involved in oxidation-reduction process, cell division, response to drug, apoptotic process, and inflammatory response in terms of molecular function (Figure 4(b)). Moreover, we also found that part of these specific PAS-associated genes (*p* < 0.05) was significantly enriched in the pathways associated with energy metabolism, such as insulin resistance, retinol metabolism, glycine, serine and threonine metabolism, Toll-like receptor signaling pathway, leishmaniasis, and folate biosynthesis (Figure 4(b)). In the above four muscle tissues, the number of PASs and gene expression were only analyzed as weak positive correlation ([Supplementary-material supplementary-material-1]).

### 3.5. Annotation of miRNAs and RBPs

**Figure 5:**
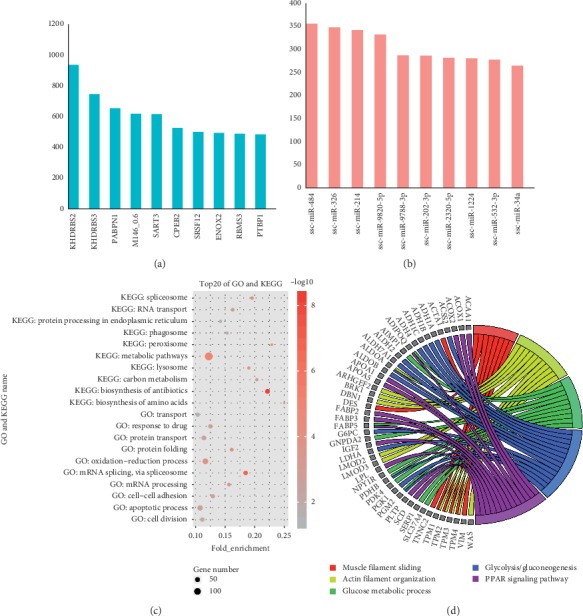
Effects of microRNAs and RNA binding proteins on APA genes. (a, b) The top 10 microRNAs and RNA binding proteins that differentially bound to the 3′ UTR ends. (c) List of first 20 pathways and biological processes about target genes of differentially microRNAs and RNA binding proteins. (d) List of 5 pathways and biological processes associated with skeletal muscle fiber transformation.

To detect possible RBPs and miRNAs involved in the regulation of gene expression and interacted with APA events, targeted binding sites for RBPs and miRNAs were searched within the 3′ UTR region. We downloaded 408 miRNA mature seed sequences from the miRBase database and 64 RBPs from CisBP-RNA database. We used 3′ UTR regions of all identified PAS-related genes to comprehensively annotate the binding sites of RBPs and miRNAs and have predicted more than 80,000 events which are listed in [Supplementary-material supplementary-material-1]. Based on APA for each target gene, we finally obtained 1149 3′ UTR regions that selectively bind to 69 miRNAs (Figure 5(a) and [Supplementary-material supplementary-material-1]). In addition, 28 RBPs positioned in 1429 3′ UTR regions with varied lengths (Figure 5(b), [Supplementary-material supplementary-material-1]). For the sake of further understanding the functions and associated pathways of these miRNAs and RBPs, we performed DAVID analysis by running queries against the DAVID database. Enrichment analysis results revealed that 1407 genes significantly (*p* < 0.05) participated in 133 biological processes (Figure 5(c)), including oxidation-reduction process, apoptotic process, protein transport, and mRNA splicing via spliceosome. 29 pathways were influenced by APA gene regulated by microRNA and RBP, respectively (Figure 5(c)). Some of these pathways and biological processes were muscle-development-related, such as muscle filament sliding, actin filament organization, glycolysis/gluconeogenesis, and PPAR signaling pathway (Figure 5(d)). Finally, we took the simultaneous existence of genes that are associated with tissue-specific PASs above and the predicted differential target genes of miRNA and RBPs. Some shared genes were discovered to be involved in muscle fiber transformation, such as HDAC (47-49), TRIB3 (50), and GYS1 [45].

### 3.6. Validation of Variable 3′ End by 3′ RACE

**Figure 6:**
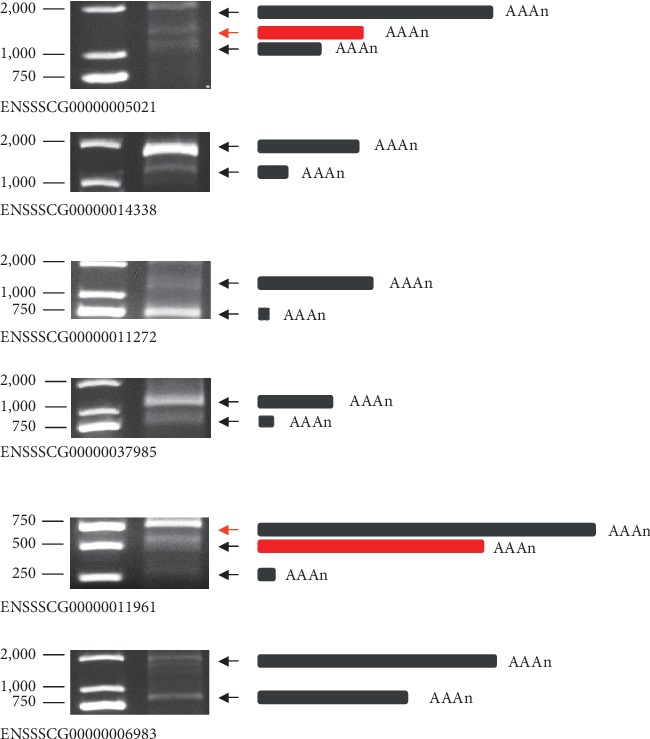
Validation of variable 3′ end by 3′ RACE. Upstream regions, solid lines. 3′ UTR, charcoal grey boxes. Unidentified PAS, red boxes.

In order to verify the accuracy of the identification and analysis of PASs, we randomly selected 6 APA genes (all PASs identified in each gene are expressed in skeletal muscle) and validated using 3′ rapid amplification of cDNA ends (3′ RACE). The results showed that the positions on the genome of all six genes were consistent with the band of 3′ RACE (Figure 6). Additional APA events were observed in the remaining two genes (ENSSSCG00000005021 and ENSSSCG00000011961), marked in red. The result of 3′ RACE can basically verify the accuracy of PASs identification and also implied that the current annotation was incomplete.

## 4. Discussion

Since the role of posttranscriptional modification has gradually gained attention, more and more studies have also focused on the polyadenylation of single gene in pig [46–48]. Many methods based on NGS technology currently emerged as the times require, including poly(A) site sequencing (PAS-seq), direct RNA sequencing (DRS), poly(A)-test RNA-sequencing (PAT-seq), and whole-transcriptome termini site sequencing (WTTS-seq) [24, 49–51]. However, there are only limited articles comprehensively annotating porcine polyadenylation with RNA-seq datasets [43]. In general, short-read RNA sequencing techniques have the fact that alignments allow us to infer, but have limitations for directly understanding the complexity of potential isoforms [22]. This study used PacBio sequencing data from 38 pooled tissues of Large White sow to identify PASs. A more comprehensive atlas of porcine PASs without assembly was established using the reported TAPIS pipeline [30]. We obtained a total of 10339 PASs which correspond to 2191 APA genes. A large part of them were distributed in the 3′ UTR regions. Although the full-length transcriptome sequencing can guarantee the reliability of the identification results, due to the insufficient sequencing depth, number of samples, and funding in this study, the quality of the PacBio dataset has limited the identification of PASs to a certain extent. Further research may need to improve the sequencing depth and analysis methods [52].

After comparing the identified PASs to the Ensembl reference annotation, we not only found that a large number of PASs are located in the intergenic regions, but also confirmed the existence of partial misannotated genes, which reflected the incompleteness of the current annotation. Single-molecule long-read sequencing technology is sufficient for the complete assembly of most known microbial genomes and has been applied to research studies of many species by now [26, 27, 30]. With the PacBio SMRT sequencing technology employment to whole-transcriptome profiling in rabbit, Chen et al. obtained 36,186 high-confidence transcripts and detected 24,797 alternative splicing (AS) and 11,184 APA events, indicating a significant improvement in the current annotation of the rabbit [26]. However, the inability of three-generation sequencing data to quantify genes makes it difficult for in-depth transcriptome study. Therefore, it is wise to combine the advantages of both sequencing methods. Gao et al. have developed a one-stop solution PRAPI that was used to perform differential expression analysis of APA through uniting PAS-seq and Iso-Seq libraries [53].

We obtained a total of 839 PASs that were mapped to the 700 intron regions of the reference genome and resulted in frame shift and subsequently produced shorter proteins. Previous studies have shown the prevalence of intron polyadenylation (IPA) in other biological processes. Singh et al. performed a comprehensive IPA analysis using 46 3′-seq and RNA-seq immune cells and identified 4927 high-reliability IPA events [54]. In this study, we found some intron PASs in TPM1, TPM2, and TPM3 (members of the tropomyosin family). Research showed the high positive correlation between TPM3 MyHC‐slow and suggested that both TPM1 and TPM3 were expressed with MyHC‐2a [55]. Further studies have shown that the synergistic expression of TPM and MyHC is an important factor in determining the contractile properties of myofibers [56]. Besides, Daloii et al. show alterations in IPA utilization in OPMD mouse and cell models. IPA may have potential regulatory mechanisms for muscle fibers development and requires further investigation.

The comparative analysis of PASs in the fast and slow muscles confirmed the existence of tissue specificity again and indicated a potential APA-dependent regulatory function. When genes with tissue-specific APA sites were functionally enriched, we found that part of genes involving in PPAR signaling pathway and insulin resistance were related to the development of fast and slow muscles. In tissue-specific analysis result, genes encoding type II HDACs (histone deacetylases) were identified with loss of the distal PAS in the slow muscle of both periods, while genes encoding type I HDACs have lost the only identified PAS in the fast muscle of newborn pigs. Previous studies showed that inhibition of MEF2 activity by class II HDAC proteins led to suppression of chronic convulsions and oxidative muscle fiber formation [57]. Galmozzi et al. explored the functional mechanism of type I HDACs and found the pivotal role of class I HDAC activity in enhancing skeletal muscle oxidative metabolism and promoting energy expenditure by increasing the expression level of PGC-1*α* [58]. Another study pinpointed that class I HDACs act as key regulators of FoxO-induced muscle atrophy during skeletal muscle disuse [59]. Our analysis also showed that the distal PAS of TRIB3 was absent in adult fast muscle. Tribbles homolog 3 (TRIB3) encodes a protein kinase of the same name and plays an important role in a variety of cellular processes, including growth and differentiation. Overexpression of TRIB3 was found to reduce PPAR-*α* activity and also increase the expression of miR499/miR208b, which led to transformation toward a slow fiber type [60]. Liu et al. revealed a downstream mechanism for muscle fiber transformation, namely, that the myosin Myh7b-encoded miR‐499 activated AMPK‐PGC‐1*α* signaling by direct inhibition to FNIP1 and thereby triggered a muscle mitochondrial oxidative metabolism program [61]. Moreover, the analysis demonstrated that PAS of CREB (cAMP response element-binding) only expressed in the adult fast muscle in our study. The CREB formed a complex with FLH3 to promote expression of a fast fiber gene and inhibited the slow fiber gene by reducing MyoD transcriptional activity [62]. On a patient with muscle-specific glycogen synthase deficiency, type I fibers predominated, glycogen deficiency and increased mitochondria, and identified homozygosity for a 2 bp deletion in the GYS1 gene [45]. The above results indicated that APA events may have an effect on muscle fiber transformation in fast and slow muscle tissues. However, some studies have shown that the difference in gene expression between adult fast and slow muscles came from the differential expression of lncRNA, not APA or AS [63]. Therefore, systematic research on fast and slow muscle development is urgent to unify different opinions based on a combination of data analysis and experimental verification of different species and developmental stages.

Since miRNA and RBP binding sites are generally located in 3′ UTRs, UTR-APA can enable isoforms to evade miRNA regulation and have significant biological effects on their translation efficiencies, stability, and localization [9, 64]. In this study, the target genes of miRNAs and RBPs were predicted systematically in the variable 3′ UTR regions, hoping to further improve the porcine conserved site annotations. Genes that differentially carry miRNAs and RBPs are enriched in muscle filament slip, actin filament tissue, PPAR signaling pathways, and glycolysis/gluconeogenesis. Interestingly, the tissue-specific genes GYS1 and TRIB3 abovementioned also combined differentially expressed miRNAs and RBPs in adult pig muscle, while HDAC2 appeared in newborn one. We predicted 7 miRNA binding sites along the altered 3′ UTR region on TRIB3, including miR-532-3p/miR-885-3p. The expression of miR-532-3p proposed to target Slc2a4 was reduced in soleus muscle from diabetic rats [65]. Massively parallel sequencing analysis of Japanese black cattle showed that miR-885 was only expressed in the semitendinosus (slow type) [66]. Among the predicted RBPs and PABPN1 proteins are known cleavage and polyadenylation associated (CPA) factors [67]. We predicted a differential binding site for PABPN1 in 3′ UTR region of RAD23A (encode UV excision repair protein RAD23 homolog A). Decreased levels of PABPN1 resulted in muscle atrophy and ECM thickening [18].

As expected, the surrounding nucleotide distribution of all identified sites was consistent with other species observations, and the AAUAAA hexamer was also regarded as the most canonical PAS in Sus scrofa within the de novo motif observation [7, 68]. In addition, we have previously observed that the proximal and distal sites in the fast and slow muscles have different usages, so we attempted to globally explore the relationship between the proximal-distal PASs and the 10 most common recognition signals in humans. The results of the study showed that the distal PAS consistent with the single-end observation was associated with a strong classical recognition signal AAUAAA, while the proximal PASs clearly carried a higher proportion of weaker variants. This may be one of the reasons for different choices of PASs in fast and slow muscles. We need to more accurately understand the molecular basis of this recognition difference by combining the protein factors directed to cleavage sites [1, 9].

## 5. Conclusions

In conclusion, our study improved and complemented the annotation of PASs and APA events in pigs by long-read sequencing. And we also found some regular patterns of recognition signals of PASs in pigs. Combining RNA-seq data for the analysis about tissue specificity and miRNAs and RBPs of APA, our results revealed that the selectivity of proximal and distal PASs possibly had potential regulation mechanism of skeletal muscle development and fiber transformation. These results not only indicated the universality of porcine polyadenylation events, provided a direction for the further study of posttranscriptional modification, and also had the positive significance for the study of fast and slow muscles in pigs.

## Data Availability

The sequence datasets of this paper have been deposited in the Genome Sequence Archive (GSA; http://gsa.big.ac.cn/) of Beijing Institute of Genomics, Chinese Academy of Sciences, with accession number PRJCA000349.

## Supplementary Materials

Supplementary Materials Supplementary file 1: Table S1: this provided the sequence of gene-specific primers for 3′RACE. Supplementary file 2: Table S2: a total of 10,334 PASs were identified according to PacBio FLNC. This provided genomic features of PASs, including located chromosome and gene, aligned FLNC reads, and location and type of PAS. 3′ UTR, 3′ untranslated regions. 5′ UTR, 5′ untranslated regions. Overlap, the location of the PAS was annotated with two overlapping genes. Intron, intron region. Intergenic, PAS located in the intergenic region of the reference annotation, predicted as a gene by our analysis. CDS, sequence coding for amino acids in protein. Error, the reference annotation gene for PAS positioning is different from our analysis. Supplementary file 3: Table S3: PASs obtained in each of the SM, EDL, BSF, and EN tissues in two periods. Supplementary file 4: Table S4: binding sites for miRNAs and RBPs were predicted for all identified 3′ UTR regions. *p* values, the optimal enrichment *p* value of the motif according to statistical tests. Adj_*p* value, *p* value was adjusted multiple times using Bonferroni correction. Here, the selection was based on the adj_*p* value <0.05. Supplementary file 5: Table S5: binding sites for miRNAs and RBPs were predicted for variable 3′UTR regions. *p* values, the optimal enrichment *p* value of the motif according to statistical tests. Adj_*p* value, *p* value was adjusted multiple times using Bonferroni correction. Here, the selection was based on the adj_*p* value <0.05. Supplementary file 6: Figure S1: Pearson correlation between gene expression and PAS number.
