# Supplementary figures and images for: Characterization and Functional Analysis of Polyadenylation Sites in Fast and Slow Muscles
Source: Biomed Res Int. 2020 Mar 16;2020:2626584. doi: 10.1155/2020/2626584 (PMC7102456; doi:10.1155/2020/2626584)

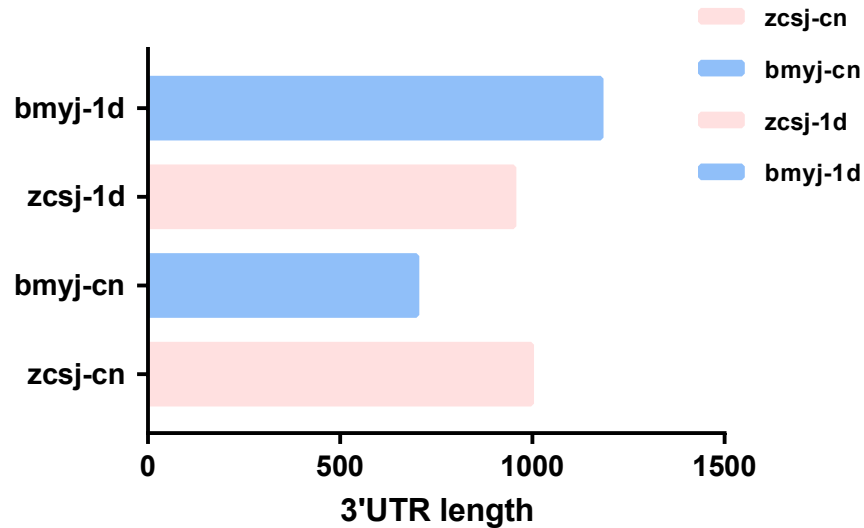

Supplement: Supplementary Materials — Supplementary file 1: Table S1: this provided the sequence of gene-specific primers for 3′RACE. Supplementary file 2: Table S2: a total of 10,334 PASs were identified according to PacBio FLNC. This provided genomic features of PASs, including located chromosome and gene, aligned FLNC reads, and location and type of PAS. 3′ UTR, 3′ untranslated regions. 5′ UTR, 5′ untranslated regions. Overlap, the location of the PAS was annotated with two overlapping genes. Intron, intron region. Intergenic, PAS located in the intergenic region of the reference annotation, predicted as a gene by our analysis. CDS, sequence coding for amino acids in protein. Error, the reference annotation gene for PAS positioning is different from our analysis. Supplementary file 3: Table S3: PASs obtained in each of the SM, EDL, BSF, and EN tissues in two periods. Supplementary file 4: Table S4: binding sites for miRNAs and RBPs were predicted for all identified 3′ UTR regions. p values, the optimal enrichment p value of the motif according to statistical tests. Adj_p value, p value was adjusted multiple times using Bonferroni correction. Here, the selection was based on the adj_p value <0.05. Supplementary file 5: Table S5: binding sites for miRNAs and RBPs were predicted for variable 3′UTR regions. p values, the optimal enrichment p value of the motif according to statistical tests. Adj_p value, p value was adjusted multiple times using Bonferroni correction. Here, the selection was based on the adj_p value <0.05. Supplementary file 6: Figure S1: Pearson correlation between gene expression and PAS number. [file 2626584.f1.zip › 2626584.f1/Supplementary Figure S1.pdf]
